# Glycoproteomics-Based Identification of Cancer Biomarkers

**DOI:** 10.1155/2011/601937

**Published:** 2011-09-28

**Authors:** Evelyn H. Kim, David E. Misek

**Affiliations:** ^1^Department of Surgery, University of Michigan Medical School, Ann Arbor, MI 48109, USA; ^2^Comprehensive Cancer Center, University of Michigan Medical School, Ann Arbor, MI 48109, USA

## Abstract

Protein glycosylation is one of the most common posttranslational modifications in mammalian cells. It is involved in many biological pathways and molecular functions and is well suited for proteomics-based disease investigations. Aberrant protein glycosylation may be associated with disease processes. Specific glycoforms of glycoproteins may serve as potential biomarkers for the early detection of disease or as biomarkers for the evaluation of therapeutic efficacy for treatment of cancer, diabetes, and other diseases. Recent technological developments, including lectin affinity chromatography and mass spectrometry, have provided researchers the ability to obtain detailed information concerning protein glycosylation. These in-depth investigations, including profiling and quantifying glycoprotein expression, as well as comprehensive glycan structural analyses may provide important information leading to the development of disease-related biomarkers. This paper describes methodologies for the detection of cancer-related glycoprotein and glycan structural alterations and briefly summarizes several current cancer-related findings.

## 1. Introduction

Within the past decade, proteomics has become an intensive field of research; one which may help to define biomarkers that could facilitate the early detection of disease or to provide important information for risk stratification, prediction of therapeutic efficacy, and disease prognosis. Proteins are known to be involved in biological activity and physiological changes in organisms [1]. Large-scale profiling of cellular proteins, using comparative expression levels between disease and normal homeostatic conditions, may reveal the basic underpinnings of disease processes. It may also facilitate the identification of proteins that are modified, either in structure or in levels of expression. Along with proteomic analysis of proteins, the analysis of protein posttranslational modifications (PTMs) also plays an important role in the study of disease. There are many types of PTMs, including acetylation, ubiquitination, phosphorylation, and glycosylation [2]. Each type of PTM may play a significant role in protein functionality. It is estimated that PTMs can be found on up to 80% of mammalian proteins [3]. Glycosylation is one of the most common PTMs, estimated to be found on over 50% of human proteins [4, 5].

**Figure 1:**
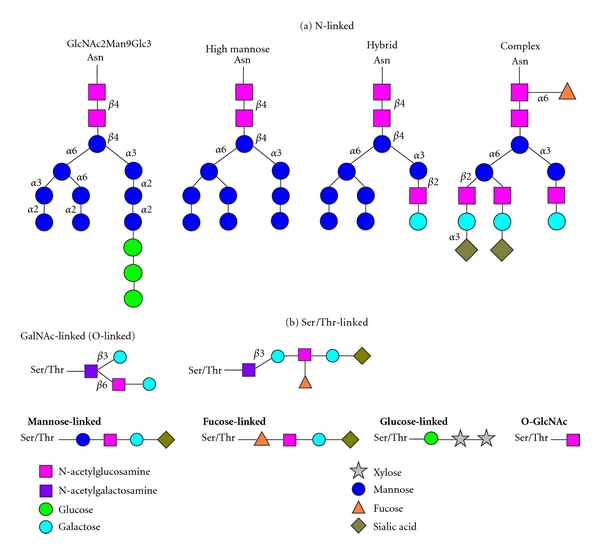
Common N- and O-linked glycans [10]. (a) Asparagine (N)-linked glycans, (b) serine/threonine (O)-linked glycans.

 Carbohydrate modifications are important in host-pathogen interactions, inflammation, development, and malignancy. Aberrant glycosylation may result in abnormal changes in biological function/activity, protein folding, and molecular recognition in disease. As such, analysis of altered cancer-related glycoprotein expression may facilitate discovery of potential biomarkers, as well as discovery of novel targets of therapeutics. Glycoproteins from various biological samples that are known to be cancer biomarkers are shown in Table 1. There are several different types of protein glycosylation, including (1) N-linked glycosylation, (2) O-linked glycosylation, (3) C-glycosylation [6], and (4) S-linked glycosylation (only found in bacteria) [7, 8]. N-glycosylation occurs on the asparagine in the sequence of Asn-X-Ser/Thr (and occasionally Cys) with X being any amino acid with the exception of proline. It is initiated on the cytoplasmic side of the rough endoplasmic reticulum (ER), where the oligosaccharide Man_5_GlcNAc_2_ is delivered to the precursor, dolichol pyrophosphate. The best known core glycan precursor is Glc_3_Man_9_GlcNAc_2_-PP-dol [9–11]. N-glycans can be further categorized by the type and position of monosaccharide residues added to the core, being either a high-mannose type, an antennary complex type, or a hybrid type (Figure 1(a) [10]). The high-mannose type of N-glycan consists of mostly mannose in the core structure. The complex type of N-glycan contains N-acetylgalactosamine (Gal*β*1-3/4GlcNAc) in the N-glycan antennal region. The antennae can be further extended by adding Gal and GlcNAc residues. The hybrid type of N-glycan has both high mannose and N-acetylglucosamine. High-mannose and hybrid types share some similar features, such as two-mannose attachment on the trimannosyl core. 

Serine or threonine residues can be O-glycosylated by addition of N-acetylglucosamine, mannose, fucose, glucose, N-acetylgalactosamine, or xylose (Figure 1(b)) [10, 11]. The most common O-linked glycosylation is initiated by N-acetylgalactosamine, bound through *α*-glycosidic linkages to Ser/Thr residues. In mucin-type O-glycosylation (mucins may be a cancer biomarker due to involvement in cancer development and influence in cell adhesion, invasion, and immune response [12]), the carbohydrate is linked to a hydroxyl group on Ser/Thr residues. This linkage often occurs while the protein is transiting through the Golgi apparatus as it is being secreted through the classical secretory pathway. The O-glycan core structure is formed by adding galactose/N-acetylglucosamine and may also contain sialic acid and/or fucose. C-glycosylation involves *α*-mannose C-linked to tryptophan. Still yet, many glycans have further modifications, such as sulfation and phosphorylation. The degree of complexity of glycan structures/composition changes, and unknown modification of glycans are beyond what can be adequately described in this paper. However, it is important to note that this complexity does contribute to cancer glycoproteomics.

## 2. Glycoproteomics Methodology

In general, glycoproteomics methodology consists of glycoprotein isolation, enrichment of the glycoproteins/glycopeptides, proteolytic digestion, and detection/identification of peptides or glycan structures using mass spectrometry-based techniques. Since many biological samples, such as plasma or serum, are very complex mixtures of proteins, extensive chromatographic separation techniques have been utilized (including ion exchange, size exclusion, hydrophobic interaction, and affinity chromatography) in order to reduce sample complexity and enhance dynamic ranges [13, 14]. Although glycoproteins can be separated by 2D-PAGE, their hydrophobic nature and tendency to precipitate at their isoelectric point, inadequate resolution, and the limited dynamic range of the gel system tend to greatly limit recovery and sequence coverage rates (by mass spectrometry). Following separation, the glycoproteins can be identified by MALDI-TOF-MS or LC-MS/MS. Recent technological improvements with LC-MS/MS have allowed mass spectrometry to play a major role in glycoproteomics analysis of disease. For glycoprotein analysis, the most commonly used methodology is a bottom-up approach [15] in which proteins are digested, after which the peptides go through an enrichment process or a deglycosylation process using Peptide N-Glycosidase F (PNGase F). PNGase F cleaves between the innermost GlcNAc and asparagine residues from N-linked glycoproteins/peptides, with the exception of ones carrying *α*1→3 linked core fucose [16]. Following digestion, the peptides are subjected to MS analysis. With MS data, bioinformatics with various algorithms and glycol-related database are heavily relied upon to analyze glycoproteins and glycans [17, 18]. UniProt and PeptideAtlas libraries [19] also can provide information of glycoproteins and glycopeptide mass spectra. Using this methodology, it is necessary to integrate obtained data about the glycan and the glycoprotein [20]. Ultimately, however, it is challenging to find disease-related glycosylation changes due to the relatively low abundance of the altered glycan/glycoprotein structures.

## 3. Glycoprotein Enrichment by Lectin Affinity Chromatography

Lectin affinity chromatographic enrichment is a routinely utilized methodology designed to concentrate glycoproteins/peptides that contain specific glycan structures, while eliminating nonspecific binding [21–24]. Various lectins (Table 2) can be used to isolate glycoproteins selectively based on glycan structure. As Concanavalin A (ConA) recognizes *α*-linked mannose residues, it will bind to high mannosyl, glycosyl, and hybrid-type glycans [25–27]. Wheat germ agglutinin (WGA) will bind to N-acetylglucosamine and possibly sialic acid residues on glycoproteins [28]. *Aleuria aurantia lectin* (AAL) recognizes specific binding to L-fucose-containing oligosaccharides [29]. Jacalin lectin (JAC) preferentially binds to galactosyl (*β*-1,3) N-acetylgalactosamine but will also bind to other O-glycosidically linked oligosaccharides (O-glycan) [30, 31]. In addition, there are many other lectins, each with their own carbohydrate binding specificity. Multiple agarose bound lectins can be used simultaneously/sequentially to purify/enrich different types of glycoproteins from various complex protein mixtures [32–34]. Utilizing multiple lectins provides the advantage of increasing detection coverage and providing global analysis. To date, however, lectin affinity chromatography has been more focused on the study of N-linked glycosylation, in part due to the fact that lectin affinity chromatography targets specific oligosaccharide structures and isolation/purification of O-linked glycan structures still in need of technological improvement. 

For the enrichment of O-linked glycosylation glycoprotein/glycopeptides, it is more common to use *β*-elimination followed by Michael addition of DTT (BEMAD) or biotinpentylamine, to label the O-glycosylation site (*O*-GlcNAc) [35]. Another method to isolate O-linked glycopeptides utilizes hydrophilic binding followed by multiple-staged MS/MS analysis [36, 37]. The detection of *O*-GlcNAc is challenging, since GlcNAcylation and phosphorylation are confined to a similar residue. One can also utilize galactosyltransferase to tag *O*-GlcNAc with ketone biotin [38]. Another extraction method, boronic acid-based beads, can provide fast, efficient, and specific enrichment of glycoproteins by binding to *cis*-diol groups on sugar residues [39, 40]. Hydrazide chemistry, interacting with glycoprotein carbonyl groups [41, 42], has also proved to be useful and is often combined with lectin affinity chromatography. Following the enrichment process, further purification with ethanol [43] or acetone [44] and a C18 stationary phase [45] or graphitized carbon column [46, 47] for glycans could be introduced prior to mass spectrometric analysis. For glycan analysis, lyophilization [48] or drying under nitrogen [49] is also useful, as increasing the temperature of the sample could cause destruction of the glycan. Further, solvent removal is very critical due to the low quantity of glycan for detection. In order to increase efficiency of isolation and detection of glycoproteins from complex protein lysates, the work flow of an online lectin microcolumn (ConA and SNA lectins) connected to LC-MS has been introduced [50]. Using silica-based columns instead of agarose-based columns, online selective concentration and detection of glycoproteins/glycopeptides gave shorter analysis time, reduced sample loss, and provided greater coverage uniformity.

## 4. Mass Spectrometry for Glycopeptides

The most widely used methods for glycomics involve characterization of glycopeptides generated by digestion and/or deglycosylation. Directly analyzing glycoproteins with attached glycans is complicated due to many fragment ions from backbone peptides, carbohydrates, and also ions from the mass spectrometry ion source. For mass analysis, there are several instruments including MALDI-TOF/TOF (time-of-flight)-MS [51–53], electrospray-based quadrupole ion trap (QIT) [54], quadrupole/TOF [55], Fourier transform ion cyclotron resonance (FTICR) [56], Orbitrap [48, 57] with CID (collision-induced dissociation), electron-capture dissociation (ECD) [58, 59], electron-transfer disassociation (ETD) [60, 61], and infrared multiphoton dissociation (IRMPD) [62, 63]. MALDI ionization generates stable singly charged precursor ions from the glycan. These precursor ions can be later characterized, using the MS/MS mode, by cleavage of glycosidic bonds and peptide with loss of glycan, leaving information regarding the glycan moiety. TOF/TOF fragmentation spectra could give additional attachment site information [64, 65] and structural analysis [66, 67]. QTOF mass spectrometers provide spectra with less chemical noise than spectra obtained by triple quadrupole or MALDI mass spectrometers. The advantages of using QTOF are higher mass accuracy, sensitivity, and resolution, thereby being able to detect ions with low intensity. Also, QTOF mass spectrometry could determine sensitive glycosylation site(s) and of the type of attached carbohydrate moiety [68]. FT-ICR-MS with ECD or IRMPD is very powerful tool for study of glycomics, since not only does FT-ICR-MS have high mass accuracy and high mass resolution, but also it has the ability to sequence peptides with no loss of glycans when it is equipped with ECD. It can also produce abundant fragment ions with IRMPD, resulting from dissociation at glycosidic linkages [57, 64].

## 5. Mass Spectrometry for Glycans

**Figure 2:**
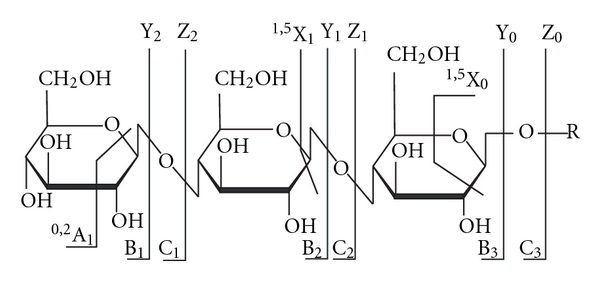
Nomenclature for tandem mass spectrometric product ions of glycans and glycoconjugated forms. Ions retaining the charge at the reducing terminus are named X, Y, and Z. Complementary ions are labeled A, B, and C [76].

N-glycan release, resulting from cleavage with PNGase F, and O-glycan release by chemical methods [69] can be detected by mass spectrometry, although it is often necessary to adapt another step to improve ionization of the glycans, such as by permethylation [70, 71] or methylation [72, 73] derivatization [74, 75]. Figure 2 shows the nomenclature for tandem mass spectrometric product ions of glycans and glycoconjugated forms [76]. Neutral glycan produces gave singly and doubly charged ions and strong signals with [M+Na]^+^, often along with [M+K]^+^ ions. Also, cross-ring and c-type fragmentation could be generated with CID. In order to increase the glycan signal, stable anionic adducts can be generated by having unstable adducts react with chloride, bromide, iodide, nitrate, and phosphate, with the products analyzed in negative mode. Occasionally, glycan analysis in negative mode provides great advantage for a strong signal, and yet still contains structural information. Acidic glycan gives ions in higher charge states due to anionic groups. It will also fragment differently due to charge localization [77]. Glycans with different isoforms from glycoconjugates can be also detected with ion mobility, since ion mobility can differ based on molecular size and shape. Fragmentation of the glycan can have several factors, such as energy level applied to ion and the charge state of the ion. There are two major ions produced, the ion from the glycosidic cleavage between sugar ring and the ion from cross-ring cleavages. Both major ions can provide linkage information of glycan.

## 6. Cancer Glycoproteomics

Aberrant protein glycosylation may result due to genetic defects, cancer, and inflammation. These changes in protein glycosylation may result in abnormal changes in biological function/activity, protein folding, and molecular recognition in cancer. The site of protein glycosylation and the structure of the oligosaccharide could be altered during initiation or progression of disease. For example, oligosaccharides such as polysialic acid, sialic acid *α*2,6-linked to galactose, *β*1-6 branching, and extended lactoseries chains (antigens) have been known to be altered in cancer [78]. There have been glycoprotein biomarker studies with identification and profiling of glycoprotein/glycan to help early diagnosis and development of new therapeutics. Current advanced technologies provide enhanced ability to detect glycoproteins/glycans with increased dynamic range and lower detection limit of analytes from complex protein lysates such as plasma [79, 80], serum [81, 82], tissues [83, 84], and bodily fluids [85, 86]. These advanced technologies also facilitate analyte quantification using labeling [87] or label-free [88] methods. There are several studies for N-linked glycoproteins associated with cancer including prostate cancer [89], ovarian cancer [90], and breast cancer [91], as well as for O-linked glycoproteins associated with disease, including prostate cancer [92] and colon cancer [93]. Also, N- and O-linked glycans have been studied for cancer biomarkers [94, 95] with glycan mass profiling, since there is alteration in the branching and differential expression of glycoforms [96]. Analysis of individual glycans may be more suitable for biomarker studies, in spite of difficulty of study due to complexity of target itself, by using specific isoform and linkage information in the future.

## 7. Glycoproteomic Analysis of Prostate Cancer 

Prostate cancer is the most common cancer in men in the United States, with an estimated 186,320 newly diagnosed cases and 28,660 deaths in 2008 [97]. Currently, the serum glycoprotein PSA is used clinically for mass population screening for prostate cancer. Unfortunately, however, assessment of PSA levels does not have the required specificity for a definitive prostate cancer diagnosis. This is due, in part, to the observed increase in expression of this protein in other prostatic pathologies such as benign prostatic hyperplasia (BPH) or prostatitis (prostate gland infection or inflammation). PSA also increases with age and infections of the prostate. Oligosaccharide profiling by mass spectrometry showed that PSA from prostate cancer sera has a higher content of *α*2,3-linked sialic acid than that from seminal fluid [98]. Lectin affinity column chromatography followed by the determination of total and free PSA by immunoassay has shown lower *α*2,6-linked sialic acid in serum free PSA from prostate cancer than that from BPH and higher *α*2,3-linked sialic acid in serum free PSA from cancer compared to BPH [99]. More recently, Meany and colleagues [100] demonstrated in a pooled sera study that *α*2,3-linked and *α*2,6-linked sialylation of PSA are more heterogeneous in cancer than in noncancer. Li and colleagues [101] monitored glycosylated and sialylated prostate-specific antigen (PSA) in prostate cancer and noncancerous tissues. They coupled a glycopeptide extraction strategy for specific glycosylation with selected reaction monitoring (SRM). Results of this study demonstrated that the relative abundance of glycosylated PSA isoforms were not correlated with total PSA protein levels measured in the same prostate cancer tissue samples by clinical immunoassay. Furthermore, the sialylated PSA was differentially distributed in cancer and noncancer tissues. These data suggest that differently glycosylated isoforms of glycoproteins can be quantitatively analyzed and may provide useful information for clinically relevant studies.

## 8. Glycoproteomics of Ovarian Cancer

Ovarian carcinoma is the leading cause of death from gynecological cancers in many Western countries. Machado et al. [90] analyzed N-linked glycans in the SKOV3 ovarian cancer cell line and on recombinant-secreted glycoprotein erythropoietin (EPO) expressed by transfected SKOV3 cells. The N-glycans were released using PNGase F and were then desalted and analyzed by high-performance anion exchange chromatography with pulsed amperometric detection and MALDI-TOF/TOF mass spectrometry. High-mannose type and fucosylated, complex type of glycans were found in the SKOV3 cancer cell line; predominant core-fucosylated structure glycans and partial LacdiNAc motif glycans were found on secreted recombinant human EPO. A large amount of N-acetylneuraminic acid in *α*-2,3-linkage was detected as were endogenous glycoproteins containing the LacdiNAc motif in N-linked glycans. The study suggests that high-mannose type glycans and LacdiNAc motif glycans may have a role as potential biomarkers for ovarian cancer. Abbott and colleagues [102] performed comparative glycotranscriptome analysis of ovarian cancer and normal ovarian tissues. Multiple lectins were utilized followed by nano-LC-linear ion trap mass spectrometry. The identified proteins were verified by immunoprecipitation and lectin blot detection. The study showed 47 potential tumor-specific lectin reactive biomarkers for ovarian cancer; periostin and thrombospondin were presented as tumor-specific glycan changes that can be used to distinguish ovarian cancer patient serum from normal serum.

N- and O-linked glycans have also been analyzed as potential ovarian cancer biomarkers [94, 95] by glycan mass profiling, since there is alteration in the branching and differential expression [103]. Bereman and colleagues [48] reported plasma glycan profiling in 10 ovarian tumors, 10 controls with a differential diagnosis of benign gynecologic tumors, and 10 healthy controls using nano-HPLC-MS using reverse phase and amide-based stationary phase column under hydrophilic interaction. The experimental data suggests that amide-based stationary phase columns may have more robustness with high mass-measurement accuracy. Results of a comparison of glycan profiling between tumor, benign tumor and normal, two fucosylated glycans showed overexpression in healthy controls. Analysis of individual glycans may be more suitable for biomarker studies, using specific isoform and linkage information in the future.

## 9. Glycoproteomics of Breast Cancer

There is growing evidence that glycan structures on glycoproteins are modified in breast cancer [104–113]. Breast-cancer-associated alterations have been demonstrated for fucosylation groups and for sialylations on the plasma protein *α*-1-proteinase inhibitor [110]. Increased GlcNAc *β*1-6Man *α*1-6Man *β*-branching in asparagine-linked oligosaccharides has been observed in human tumor cells. The levels of the *β*1-6 branched oligosaccharides were evaluated in a series of benign and malignant human breast biopsies. Normal human breast tissue and benign lesions showed low expression, but 50% of the primary malignancies examined showed significantly elevated *β*1-6 branching [111]. Subsequently, L-PHA (a lectin that binds specifically to the *β*1-6 branched oligosaccharides) lectin histochemistry was performed on paraffin sections of human breast tissues. All breast carcinomas and epithelial hyperplasia with atypia demonstrated significantly increased L-PHA staining as compared to fibroadenomas and hyperplasia without atypia [112]. More recently, L-PHA reactive glycoproteins were identified from matched normal (nondiseased) and malignant tissue isolated from patients with invasive ductal breast carcinoma [113]. Comparison analysis of the data identified 34 proteins that were enriched by L-PHA fractionation in tumor relative to normal tissue for at least 2 cases of ductal invasive breast carcinoma. Of these 34 L-PHA tumor enriched proteins, 12 were common to all 4 matched cases analyzed.

Rudd and coworkers [114] analyzed fluorescently tagged serum *N*-glycans of advanced breast cancer patients using exoglycosidases and LC-MS/MS. They found that the expression of a trisialylated triantennary glycan containing an *α*-1,3-linked fucose was increased in the presence of breast cancer. Novotny and coworkers profiled the permethylated *N*-glycans in sera of breast cancer patients at different stages (stages I to IV) using MALDI TOF/TOF MS in one study [115]. In a second study, they profiled reduced and methylated serum *N*-glycans of late-stage breast cancer patients using nanoliquid chromatography (LC) Chip/time-of-flight (TOF) MS [116]. In both studies, they found an increase in fucosylation in both core and branched segments of *N*-glycans in the presence of breast cancer. In the latter study, they found a decrease in expression of a biantennary-monosialylated *N*-linked glycan and an increase in expression of a fucosylated triantennary-trisialylated *N*-linked glycan in the presence of stage IV breast cancer. These glycosylation changes in a tumor-secreted protein may reflect fundamental activity changes in the enzymes involved in the glycosylation pathway, either through altered levels of enzymes or altered enzymatic activity. Importantly, the changes in glycan structure may serve as early detection biomarkers of breast cancer.

## 10. Summary

Glycoproteomic analysis has become an important part of proteomics because not only does glycosylation reveal biological changes in terms of disease, but also it is possible to study glycoprotein/glycan in depth with the recent development of new mass spectrometry technology. For isolation and detection of glycoprotein/glycopeptides and glycans, an enrichment process using affinity chromatography followed by mass spectrometry with MS/MS can be used with quantitation. There is still massive, intense, manual data processing for studying glycan structure analysis, however, bioinformatics with various algorithms and glycol-related database has been developed to help analyze glycoprotein and glycan. The study of glycoproteomics is technically challenging yet has begun to produce promising results to identify biomarkers for early diagnosis and disease therapeutics.

## Figures and Tables

**Table 1:** List of some of the US Food and Drug Administration (FDA) approved glycoprotein cancer biomarkers. CA: cancer antigen, FDP: fibrin degradation protein, sPIgR: secreted chain of the polymeric immunoglobulin receptor.

Biomarker(a)	Glycosylation	Source	Disease
CA15.3	Yes	Serum	Breast
CA27-29	Yes	Serum	Breast cancer
HER2/NEU	Yes	Serum	Breast cancer
Fibrin/FDP	Yes	Urine	Bladder
CEA (carcinoembryonic antigen)	Yes	Serum	Colon cancer
Carcinoembryonic antigen (CEA)	Yes	Serum	Colon, breast, lung, pancreatic
Epidermal growth factor receptor	Yes	Tissue	Colon cancer
CA19-9	Yes	Serum	Gastrointestinal
KIT	Yes	Tissue	Gastrointestinal tumor
*α*-fetoprotein(AFP)	Yes	Serum	Hepatoma, testicular cancer
Human chorionic gonadotropin-*β*	Yes	Serum	Testicular cancer
Thyroglobulin	Yes	Serum	Thyroid cancer
CA125	Yes	Serum	Ovarian
PSA (prostate-specific antigen)	Yes	Serum	Prostate

**Table 2:** A partial list of lectins commonly used for enrichment of glycoproteins/glycopeptides.

Lectin	Specificity
Aleuria Aurantia Lectin (AAL)	Fuc*α*1-6 GlcNAc, Fuc*α*1-3(Gal*β*1-4)GlcNAc
Concanavalin A (Con A)	High-Mannose, Man*α*1-6(Man*α*1-3)Man
Erythrina Cristagalli Lectin (ECA)	Gal*β*1-4GlcNAc
Hippeastrum Hybrid Lectin (HHL, AL)	High-Mannose, Man*α*1-3Man, Man*α*1-6Man
Jacalin	Gal*β*1-3GalNAc, GalNAc
Lens Culinaris Agglutinin (LCA)	Fuc*α*1-6 GlcNAc, *α*-D-Glc, *α*-D-Man
Maackia Amurensis Lectin (MAL)	Sia*α*2-3Gal*β*1-4GlcNAc
Peanut Agglutinin (PNA)	Gal*β*1-3GalNAc
Phaseolus vulgaris Leucoagglutinin (PHA-L)	Tri/tetra-antennary complex-type N-glycan
Sambucus Nigra Lectin (SNA, EBL)	Sia*α*2-6Gal/GalNAc
Ulex Europaeus Agglutinin-I (UEA-I)	Fuc*α*1-2Gal*β*1-4GlcNAc
Wheat Germ Agglutinin (WGA)	Chitin oligomers, Sia
